# Genetic Overlap between Apparently Sporadic Motor Neuron Diseases

**DOI:** 10.1371/journal.pone.0048983

**Published:** 2012-11-14

**Authors:** Marka van Blitterswijk, Lotte Vlam, Michael A. van Es, W-Ludo van der Pol, Eric A. M. Hennekam, Dennis Dooijes, Helenius J. Schelhaas, Anneke J. van der Kooi, Marianne de Visser, Jan H. Veldink, Leonard H. van den Berg

**Affiliations:** 1 Department of Neurology, Rudolf Magnus Institute of Neuroscience, University Medical Center Utrecht, Utrecht, The Netherlands; 2 Department of Medical Genetics, University Medical Center Utrecht, Utrecht, The Netherlands; 3 Department of Neurology, Donders Institute for Brain, Cognition and Behavior, Radboud University Nijmegen Medical Center, Nijmegen, The Netherlands; 4 Department of Neurology, Academic Medical Center, University of Amsterdam, Amsterdam, The Netherlands; Lousiana State University Health Sciences Center, United States of America

## Abstract

Progressive muscular atrophy (PMA) and amyotrophic lateral sclerosis (ALS) are devastating motor neuron diseases (MNDs), which result in muscle weakness and/or spasticity. We compared mutation frequencies in genes known to be associated with MNDs between patients with apparently sporadic PMA and ALS. A total of 261 patients with adult-onset sporadic PMA, patients with sporadic ALS, and control subjects of Dutch descent were obtained at national referral centers for neuromuscular diseases in The Netherlands. Sanger sequencing was used to screen these subjects for mutations in the coding regions of superoxide dismutase-1 (*SOD1*), angiogenin (*ANG*), fused in sarcoma/translated in liposarcoma (*FUS/TLS*), TAR DNA-binding protein 43 (*TARDBP*), and multivesicular body protein 2B (*CHMP2B*). In our cohort of PMA patients we identified two *SOD1* mutations (p.D90A, p.I113T), one *ANG* mutation (p.K17I), one *FUS/TLS* mutation (p.R521H), one *TARDBP* mutation (p.N352S), and one novel *CHMP2B* mutation (p.R69Q). The mutation frequency of these genes was similar in sporadic PMA (2.7%) and ALS (2.0%) patients, and therefore, our findings demonstrate a genetic overlap between apparently sporadic PMA and ALS.

## Introduction

Motor neuron diseases (MNDs) are a heterogeneous group of disorders characterized by muscle weakness and/or spasticity due to degeneration of motor neurons. Progressive muscular atrophy (PMA) refers to a subgroup of the MND patients with rapidly or gradually developing muscle weakness. PMA accounts for 5–10% of adult-onset MNDs, and is caused by a progressive loss of lower motor neurons (LMNs) [1], [2]. Differentiation of PMA from amyotrophic lateral sclerosis (ALS) is important, since the median survival of patients with PMA is significantly longer than that of patients with ALS [3].

The etiology of MNDs is complex. Most of the ALS cases, for instance, are sporadic in nature and thought to be caused by an interaction of genetic and environmental factors [4]. Currently, many genes appear to be involved in the pathogenesis of ALS, including chromosome 9 open reading frame 72 (*C9orf72*), superoxide dismutase-1 (*SOD1*), angiogenin (*ANG*), fused in sarcoma/translated in liposarcoma (*FUS/TLS*), TAR DNA-binding protein 43 (*TARDBP/*TDP-43), vesicle-associated membrane protein B (*VAPB*), optineurin (*OPTN*), valosin-containing protein (*VCP*), ubiquilin-2 (*UBQLN2*), sequestosome-1 (*SQSTM1*), and profilin-1 (*PFN1*) [5], [6], [7], [8], [9], [10], [11]. We have recently shown that *C9orf72* repeat expansions can also be detected in apparently sporadic PMA, but at a lower frequency (1.6%) than in apparently sporadic ALS (6.1%) [12]. Moreover, we have demonstrated that mutations in four major MND-associated genes, *SOD1*, *ANG*, *FUS/TLS* and *TARDBP*, account for less than two percent of the sporadic ALS cases [13]. The combined mutation frequency of these four MND-associated genes is unknown for sporadic PMA patients. Mutations in charged multivesicular body protein 2B (*CHMP2B*) have, however, been reported in sporadic PMA patients [14], [15].

The objective of this study is to determine the mutation frequency of major MND-associated genes in patients with apparently sporadic PMA. We compared their mutation frequencies to those in a large cohort of patients with apparently sporadic ALS, revealing a genetic overlap.

## Materials and Methods

### Patient Selection

We included 261 patients with apparently sporadic PMA and screened their DNA for mutations in *SOD1*, *ANG*, *FUS/TLS*, *TARDBP*, and *CHMP2B*. PMA patients had already been screened for mutations in transient receptor potential vanilloid 4 (*TRPV4*) and repeat expansions in *C9orf72* [12], [16]. Their diagnosis was based on LMN involvement on clinical and electrophysiological examination at time of referral. We excluded patients with a family history of PMA, a history of acute poliomyelitis, spinal radiculopathy, diabetic amyotrophy, thyrotoxicosis, or hyperparathyroidism, clinical signs of upper motor neuron (UMN) involvement, sensory signs on neurological examination, structural lesions on magnetic resonance imaging or computed tomography of head and spine, and motor conduction block on extensive standardized nerve conduction studies [2].

Cohorts of sporadic ALS patients had already been screened for mutations in *SOD1* (N = 451), *ANG* (N = 941), *FUS/TLS* (N = 1,192), *TARDBP* (N = 1,192), and *C9orf72* (N = 1,422) [12], [13], [17], [18], [19]. We screened 1,002 sporadic ALS patients for mutations in *CHMP2B*. ALS patients were recruited through the Dutch Prospective Population-based ALS registry; they were diagnosed according to the El Escorial Criteria at national referral centers for neuromuscular diseases (University Medical Center Utrecht, Academic Medical Center Amsterdam, or Radboud University Nijmegen Medical Center) [20], [21].

Mutations in *SOD1* (N = 1,894), *ANG* (N = 1,582), *FUS/TLS* (N = 970), and *TARDBP* (N = 1,415) had previously been reported in Dutch control subjects [13], [18], [19]. We screened a total of 750 control subjects of Dutch descent for mutations in *CHMP2B*.

### Ethics Statement

All material was obtained with approval of the medical ethics committee for research in humans of the University Medical Center Utrecht, The Netherlands, and all participants gave written informed consent.

### Genetic Analysis

Coding regions of *SOD1* (NM_000454.4), *ANG* (NM_001145.4), *FUS/TLS* (NM_004960.3, exon 5, 6, 14, 15), *TARDBP* (NM_007375.3, exon 6), and *CHMP2B* (NM_014043.3) were screened for mutations using touchdown PCR, as described previously [13], [22]. Sanger sequencing and data analysis were performed with BigDye Terminator 3.1 sequencing kit (Applied Biosystems, Foster City, California), DNA Analyzer 3730XL (Applied Biosystems) and PolyPhred [23]. Each mutation was confirmed on genomic DNA and its impact on the structure, and function of the protein was predicted with PolyPhen-2 (PolyPhen-2 version 2.1.0; http://genetics.bwh.harvard.edu/pph2/) and PMut (http://mmb.pcb.ub.es/PMut/PMut.jsp).

### Genealogical Analysis

Lists of descendants were compiled for index patients. Based on these lists, civil records/registers, and church records of the Dutch population, pedigrees were generated (containing two parents, four grandparents, eight great-grandparents, etc.). This information was then used to determine whether index patients were related, and detailed family trees were constructed.

### Haplotype Analysis

Extended haplotype analysis, using six extragenic polymorphic markers flanking *TARDBP* (D1S1612, D1S503, D1S244 proximal of *TARDBP,* and D1S2667, D1S2740 and D1S1597 distal of *TARDBP*), was performed to construct a haplotype segregating with the identified p.N352S mutation in *TARDBP*. Validity of the constructed haplotype was determined by segregation analysis in families and patients whose DNA was available for testing.

### Statistical Analysis

A Fisher’s exact test or Chi-square test was used to compare mutation frequencies, gender, site of onset, and current status (alive/deceased) between PMA and ALS patients; a Mann-Whitney test was used to compare age at onset and disease duration (GraphPad Prism version 5; http://www.graphpad.com). P-values below 0.05 were considered significant.

## Results

### Study Population

Baseline characteristics of the 261 sporadic PMA patients and 1,002 sporadic ALS patients are shown in Table 1. Patients with PMA were more likely to be male (72% versus 59%); furthermore, they lived longer (7.6 year versus 3.8 year), and had a lower age at onset (58.0 year versus 60.6 year) than patients with ALS.

**Table 1:** Baseline characteristics of study population.

Cohort	Number (N)	Male/female (N) (%)	Age at onset (y) (CI)	Alive/deceased (N) (%)	Duration (y) (CI)
**PMA**	261	187/74 (72/28)	58.0 (56.4–59.7)	137/116 (54/46)	7.6 (6.7–8.5)
**ALS**	1,002	593/409 (59/41)	60.6 (59.8–61.3)	135/854 (14/86)	3.8 (3.6–4.1)

Abbreviations: PMA = progressive muscular atrophy, ALS = amyotrophic lateral sclerosis, N = number, y = years, and CI = 95% confidence interval. Disease duration is defined as the interval between age at onset and age at death, or between age at onset and age last known to be alive. Patients with sporadic PMA are more likely to be male (p-value 0.001), to have a lower age at onset (p-value 0.010), to be alive (p-value <0.001), and to have a longer disease duration than patients with sporadic ALS (p-value <0.001).

### Mutation Frequencies

Table 2 summarizes the mutations found in patients and control subjects. In individual PMA patients we detected heterozygous mutations in *SOD1* (p.D90A [c.1078A>C], p.I113T [c.1147T>C]), *ANG* (p.K17I [c.122A>T]), *FUS/TLS* (p.R521H [c.1562G>A]) and *TARDBP* (p.N352S [c.1055A>G]), accounting for 2.3% of the patients. Previously, we showed that missense mutations in *SOD1*, *ANG*, *FUS/TLS* and *TARDBP* were present in 1.7% of the ALS patients, and 0.4% of the control subjects [13], [17], [18], [19]. In our current study, we also identified four novel *CHMP2B* mutations, one of which was present in a PMA patient (p.R69Q [c.206G>A]), and three in ALS patients (p.R22Q [c.65G>A], p.N54T [c.161A>C], p.T83I [c.248C>T]). All four *CHMP2B* mutations are located in a domain that is important for the formation of multivesicular bodies (MVBs), involved in sorting of cargo proteins to intraluminal vesicles [24]. These mutations are located in well conserved areas (Figure S1) and predicted to be pathological (Table 2). They account for 0.38% of the sporadic PMA patients and 0.30% of the sporadic ALS patients. None of these *CHMP2B* mutations was present in our control subjects; however, in one control subject (0.13%) we did detect a mutation (p.S194L [c.581C>T]) that had previously been reported in a patient with frontotemporal dementia (FTD) [25].

**Table 2:** Missense mutations found in *SOD1, ANG, FUS/TLS, TARDBP,* and *CHMP2B*.

Gene	Variant	Exon	PMA	ALS	CON	Prediction PolyPhen-2	Prediction PMut
*SOD1*	p.D90A	4	1/261^a^	1/451 [17]	3/1,894 [13]	Benign	Pathological
	p.I113T	4	1/261	0/451	0/1,894	Probably damaging	Pathological
	p.I99V	4	0/261	1/451	0/1,894	Benign	Neutral
	**Total (%)**		**2/261 (0.77)**	**2/451 (0.44)**	**3/1,894 (0.16)**		
*ANG*	p.G(−10)D	2	0/261^a^	1/941 [18]	0/1,582 [18]	N/A	N/A
	p.K17I	2	1/261	3/941	2/1,582	Benign	Pathological
	p.T80S	2	0/261	1/941	0/1,582	Possibly damaging	Neutral
	p.F100I	2	0/261	1/941	0/1,582	Probably damaging	Neutral
	**Total (%)**		**1/261 (0.38)**	**6/941 (0.64)**	**2/1,582 (0.13)**		
*FUS/TLS*	p.S115N	5	0/261^a^	1/1,192 [13]	0/970 [19]	Unknown	Neutral
	p.Q210H	6	0/261	0/1,192	1/970	Unknown	Neutral
	p.R487C	14	0/261	1/1,192	0/970	Probably damaging	Pathological
	p.R495X	14	0/261	1/1,192	0/970	N/A	N/A
	p.R521H	15	1/261	0/1,192	0/970	Probably damaging	Pathological
	**Total (%)**		**1/261 (0.38)**	**3/1,192 (0.17)**	**1/970 (0.10)**		
*TARDBP*	p.N352S	6	2/261^a^	3/1,192 [13]	0/1,415 [13]	Benign	Pathological
	p.I383V	6	0/261	1/1,192	0/1,415	Benign	Neutral
	**Total (%)**		**2/261 (0.77)**	**4/1,192 (0.34)**	**0/1,415 (0.00)**		
*CHMP2B*	p.R22Q	2	0/261^a^	1/1,002^a^	0/750^a^	Possibly damaging	Pathological
	p.N54T	3	0/261	1/1,002	0/750	Probably damaging	Neutral
	p.R69Q	3	1/261	0/1,002	0/750	Probably damaging	Pathological
	p.T83I	3	0/261	1/1,002	0/750	Probably damaging	Pathological
	p.S194L	6	0/261	0/1,002	1/750	Benign	Neutral
	**Total (%)**		**1/261 (0.38)**	**3/1,002 (0.30)**	**1/750 (0.13)**		
	**Total (%)**		**7 (2.7)**	**18 (2.0)**	**7 (0.5)**		

Abbreviations: CON = control subjects, and N/A = not applicable. Mutations in *SOD1*, *ANG*, *FUS/TLS*, *TARDBP,* and *CHMP2B* were present in 2.7% of the PMA patients, 2.0% of the ALS patients, and 0.5% of the control subjects. No PMA patients were detected with mutations in multiple MND-associated genes. A Fisher’s exact test or Chi-square test was used to compare mutation frequencies between patients with PMA and ALS for each gene; no significant differences were detected (data not shown for simplicity).

a Cohort described in the present study.

### Clinical Characteristics

The average age at onset of PMA patients with missense mutations was 48 years, and five of them were male (71%). Although only one of these patients had died, their average disease duration already exceeded 114 months (range 37–316). These clinical characteristics of sporadic PMA patients with missense mutations were consistent with the characteristics of our entire PMA cohort. More detailed signs and symptoms are provided in Table 3.

**Table 3:** Clinical characteristics of newly identified patients with missense mutations.

Group	Gene	Variant	Gender	LMN^a^ signs	UMN^a^ signs	Age at onset (y)	Site of onset	Duration (m)
**PMA**	*SOD1*	p.D90A	M	1	0	17	Cervical	316
		p.I113T	F	2	0	48	Lumbosacral	108
	*ANG*	p.K17I	M	1	0	66	Lumbosacral	52
	*FUS/TLS*	p.R521H	M	3	0	47	Cervical	68
	*TARDBP*	p.N352S	F	2	0	68	Cervical	37
		p.N352S	M	4	0	61	Lumbosacral	101^b^
	*CHMP2B*	p.R69Q	M	1	0	26	Cervical	116
**ALS**	*CHMP2B*	p.R22Q	M	3	2	57	Cervical	68
		p.N54T	F	3	2	68	Bulbar	28^b^
		p.T83I	M	2	1	71	Cervical	75

Abbreviations: M = male, F = female, LMN = lower motor neuron, UMN = upper motor neuron, and m = months. Clinical characteristics of ALS patients with *SOD1*, *ANG*, *FUS/TLS* and *TARDBP* mutations have been described elsewhere [13], [17], [18], [19].

a Number of affected body regions at time of diagnosis (maximum four: bulbar, cervical, thoracic or lumbosacral).

b Deceased.

### Genealogical- and Haplotype Analyses

Previously, we have shown that the p.N352S mutation in *TARDBP* is a founder mutation in the Dutch ALS population [13]. Hence, we performed a thorough genealogical analysis and demonstrated that our PMA patients with p.N352S mutations had common ancestors, dating back to the 17^th^ century in the north of France (Figure S2). Haplotype analysis revealed that these patients also shared the haplotype that was reported in Dutch ALS patients [13].

## Discussion

Patients with isolated LMN signs represent a subgroup of the patients with MND. To assess the mutation frequency of MND-associated genes in this subgroup, we compared 261 apparently sporadic PMA patients to apparently sporadic ALS patients. Our PMA patients were more likely to be male and lived significantly longer than ALS patients, as reported previously [3]. We detected two *SOD1* mutations (p.D90A, p.I113T), one *ANG* mutation (p.K17I), one *FUS/TLS* mutation (p.R521H), one *TARDBP* mutation (p.N352S), and one novel *CHMP2B* mutation (p.R69Q) in individual PMA patients. For each of these genes we compared mutation frequencies between our PMA patients and ALS patients, and did not detect significant differences.

Clinical and pathological similarities between PMA and ALS have already been reported: more than twenty percent of the patients with isolated LMN signs will develop UMN signs within six years, especially in the first years after symptom onset [1], [3], [26]. Nonetheless, it can be difficult to diagnose these UMN signs due to LMN wasting and pathophysiological abnormalities caused by damaged motor pathways, motor neurons and interneurons [27]. Pathological studies have also revealed ubiquitinated inclusions and involvement of the corticospinal tract in PMA patients, which are typical for ALS patients and emphasize similarities between these diseases [28], [29].

Previously, mutations in *SOD1*, *ANG*, *FUS/TLS*, *TARDBP* and *CHMP2B* have been identified in patients with a range of clinical phenotypes, including combinations of FTD, Parkinson’s disease, and ALS [14], [18], [22], [30], [31], [32], [33], [34], [35], [36], [37], [38], [39], [40], [41], [42], [43], [44], [45]. *CHMP2B* mutations have also been described in sporadic PMA patients, while mutations in MND-associated genes have been detected in familial ALS patients with predominantly LMN signs [15], [19], [28], [46], [47], [48], [49], [50]. In addition, we have recently shown that *C9orf72* repeat expansions are present in sporadic PMA patients, but at a lower frequency than in sporadic ALS patients (1.6% versus 6.1%) [12]; other studies have shown that *C9orf72* repeat expansions were present in approximately 7% of white sporadic ALS patients from the USA, Europe and Australia, and that bulbar onset ALS is frequently encountered in patients with these expansions [51], [52], [53], [54]. Our current findings demonstrate that mutations in *SOD1*, *ANG*, *FUS/TLS*, *TARDBP* and *CHMP2B* are also associated with apparently sporadic PMA, thus expanding the wide range of clinical phenotypes reported to date. Furthermore, the comparable mutation frequencies between PMA and ALS patients show that, apart from clinical and pathological similarities, these diseases demonstrate a genetic overlap as well, suggesting that PMA is a subtype of ALS.

We detected a *SOD1* mutation (p.D90A) in a patient with sporadic PMA, a patient with classical sporadic ALS, and control subjects. This is the most common *SOD1* mutation, and causes both autosomal dominant and recessive ALS [55], [56]. Although it behaves dominantly in many families, it is a polymorphism in the Swedish population, primarily causing ALS when in the homozygous state [57]. Another *SOD1* mutation (p.I113T) was also present in a PMA patient; it is known for its clinical heterogeneity, including asymptomatic subjects, patients with mild fasciculations, patients with typical ALS, and patients with ALS-FTD and chorea [41], [58]. Both these *SOD1* mutations appear to result in ALS through aggregation of mutant SOD1 protein [59].

In addition, we identified an *ANG* mutation (p.K17I) in one PMA patient, and in two out of 1,582 Dutch control subjects. The p.K17I mutation has already been reported in ALS patients and in control subjects [18]. Despite its presence in control subjects, it does affect the neuroprotective-, angiogenic- and ribonucleolytic activity of ANG [50], [60], [61]. It seems likely that this mutation raises ALS susceptibility and/or acts as a genetic modifier, a hypothesis supported by recent reports of families that harbor a p.K17I mutation in combination with *TARDBP*- or *FUS/TLS* mutations, and a large international collaborative study, which demonstrates that *ANG* mutations confer a substantial risk for ALS [13], [18], [62].

In one PMA patient, we identified a *FUS/TLS* mutation (p.R521H); one of the most common *FUS/TLS* mutations with a disease duration of approximately four years [63], [64]. In two other PMA patients we detected a *TARDBP* mutation (p.N352S), which has been described in German and Japanese ALS patients [65], [66], [67]. We have recently reported that p.N352S is a founder mutation in the Dutch ALS population [13]. In the present study, we revealed that our PMA patients had common ancestors and shared a haplotype also detected in Dutch ALS patients.

The four *CHMP2B* mutations we detected (p.R69Q, p.R22Q, p.N54T, p.T83I) are novel, absent in control subjects, located in well conserved areas, and predicted to be pathogenic. One of these was identified in a patient with sporadic PMA (0.38%), three in patients with sporadic ALS (0.30%). These mutation frequencies demonstrate that *CHMP2B* mutations are not specific for PMA, but are present in patients with PMA, FTD, ALS-FTD, and ALS. We also detected one previously reported mutation (p.S194L) in a control subject [25]. Since this variant is located within an area of low complexity and predicted to have neutral effects, it probably represents a rare benign polymorphism.

Recently, we have provided evidence for an oligogenic etiology of familial ALS [13]. We reported five families with mutations in multiple MND-associated genes: *ANG* mutations were detected in combination with *FUS/TLS* and *TARDBP* mutations, and *C9orf72* repeat expansions in combination with *TARDBP*, *SOD1*, and *FUS/TLS* mutations (p-value 1.57×10^−7^). In our present study, we did not identify mutations in multiple MND-associated genes in a single patient; however, the high phenotypic variability that is seen amongst patients with mutations in these genes (ranging from FTD to Parkinson’s disease and MNDs) does suggest that other genetic and/or environmental factors influence disease characteristics of sporadic MNDs.

To summarize, we have detected comparable mutation frequencies in patients with apparently sporadic PMA and ALS, indicating a genetic overlap between these two diseases. Thus, our findings favor the hypothesis that PMA is a subtype of ALS and not a distinct entity, broadening the disease spectrum of ALS.

## Supporting Information

Figure S1 ***CHMP2B*** **mutations and conservation.** Conservation of amino-acid residues across species was generated using ClustalW2 online tool, http://www.ebi.ac.uk/Tools/msa/clustalw2/.
(DOC)

Figure S2 **Pedigree of two PMA patients with p.N352S mutations in** ***TARDBP*** **.**
(DOC)
