# Supplementary material for: Genetic Overlap between Apparently Sporadic Motor Neuron Diseases
Source: PLoS One. 2012 Nov 14;7(11):e48983. doi: 10.1371/journal.pone.0048983 (PMC3498376; doi:10.1371/journal.pone.0048983)
Supplement: Figure S1 — CHMP2B mutations and conservation. Conservation of amino-acid residues across species was generated using ClustalW2 online tool, http://www.ebi.ac.uk/Tools/msa/clustalw2/. (DOC) [file pone.0048983.s001.doc]

**Figure S1.** *CHMP2B* mutations and conservation.

p.R22Q

p.N54T

p.T83I

p.R69Q


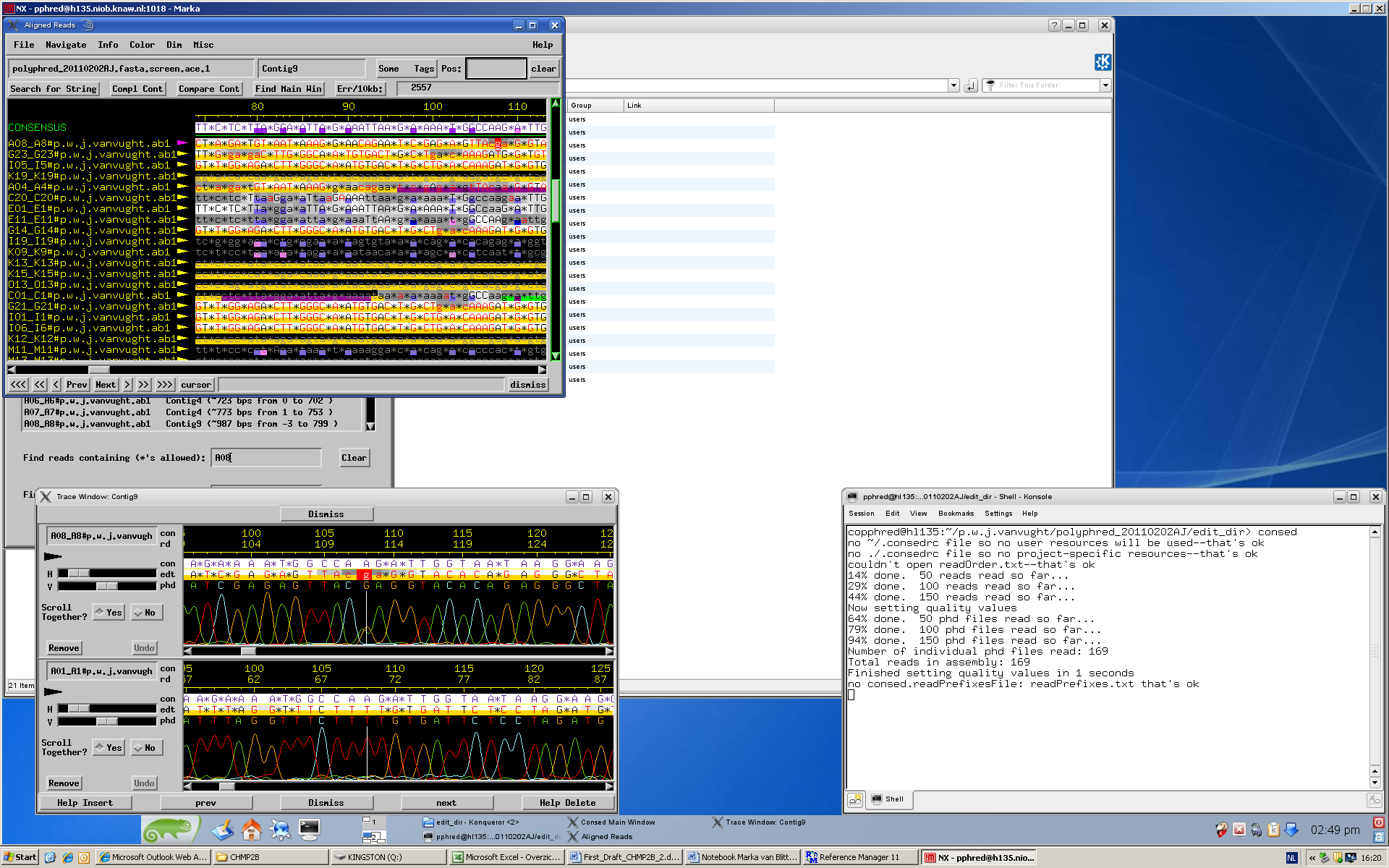

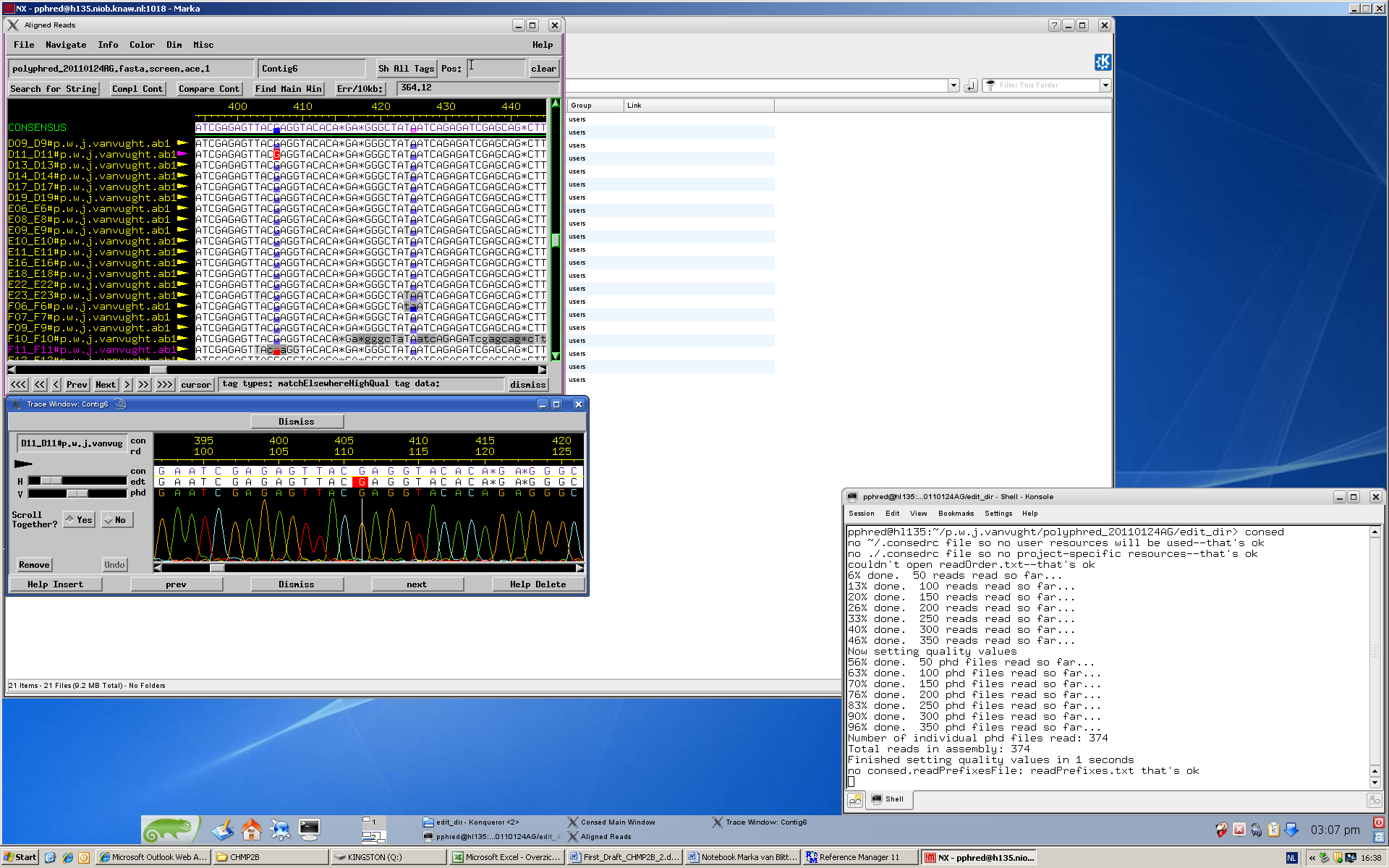

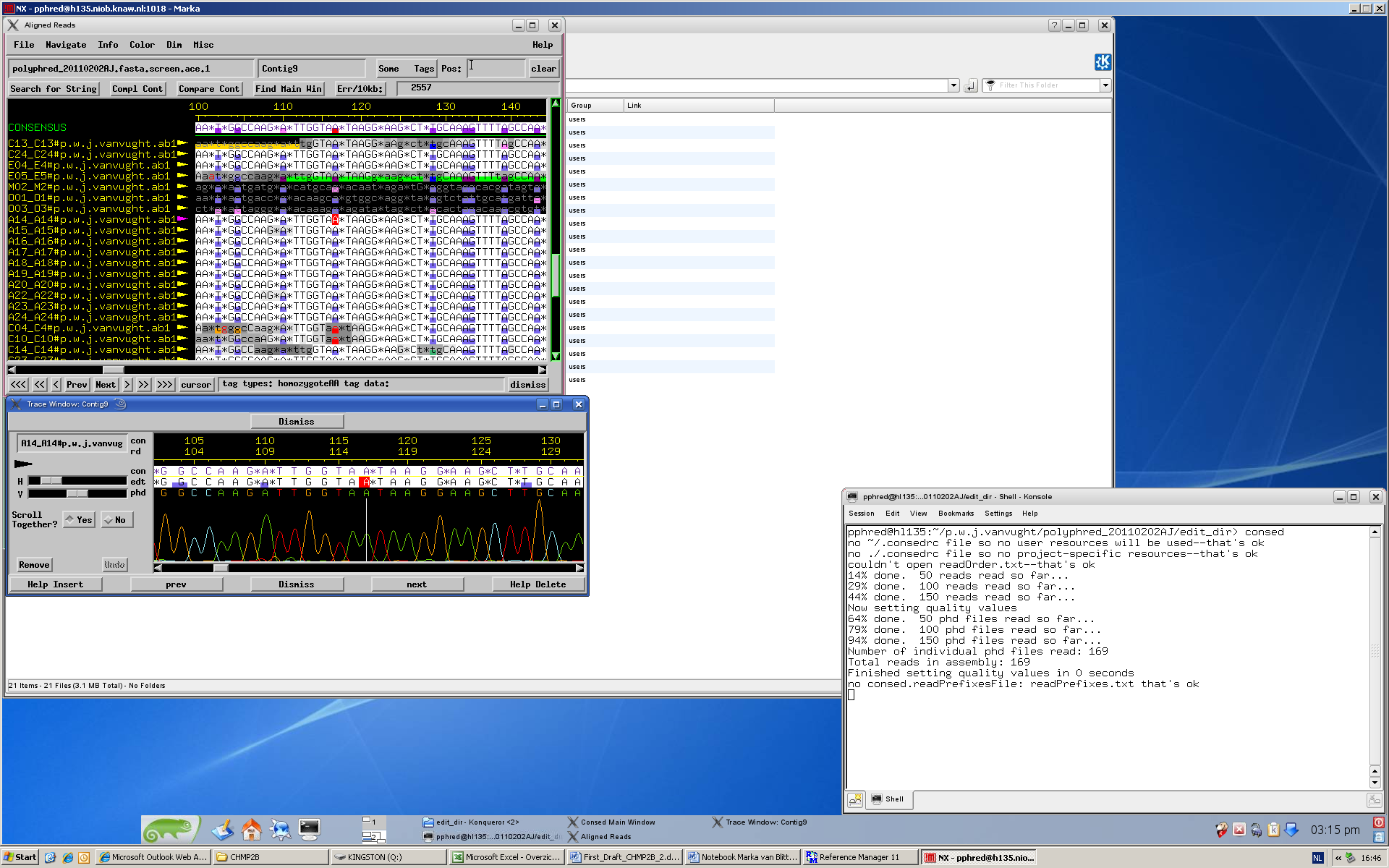

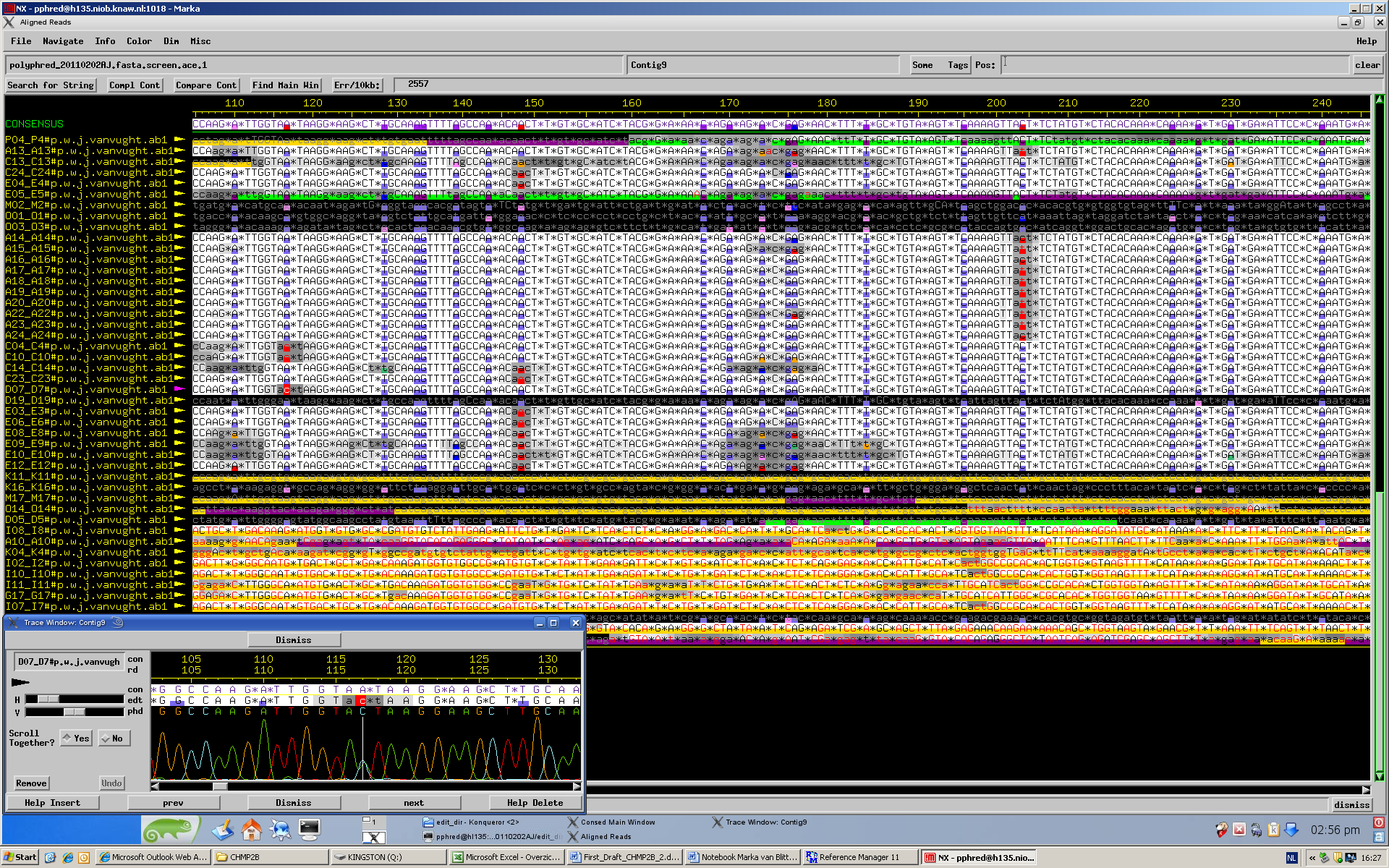

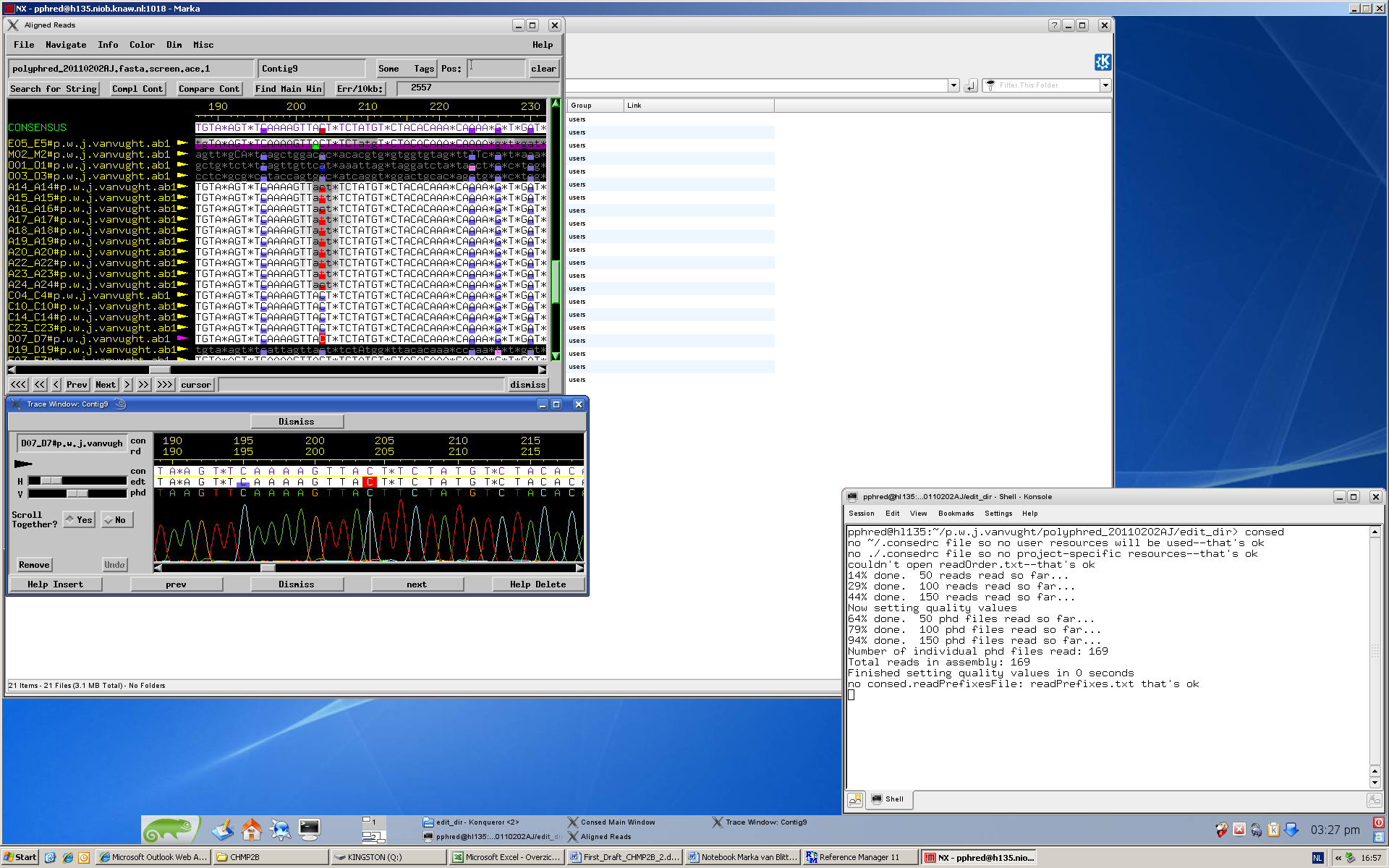

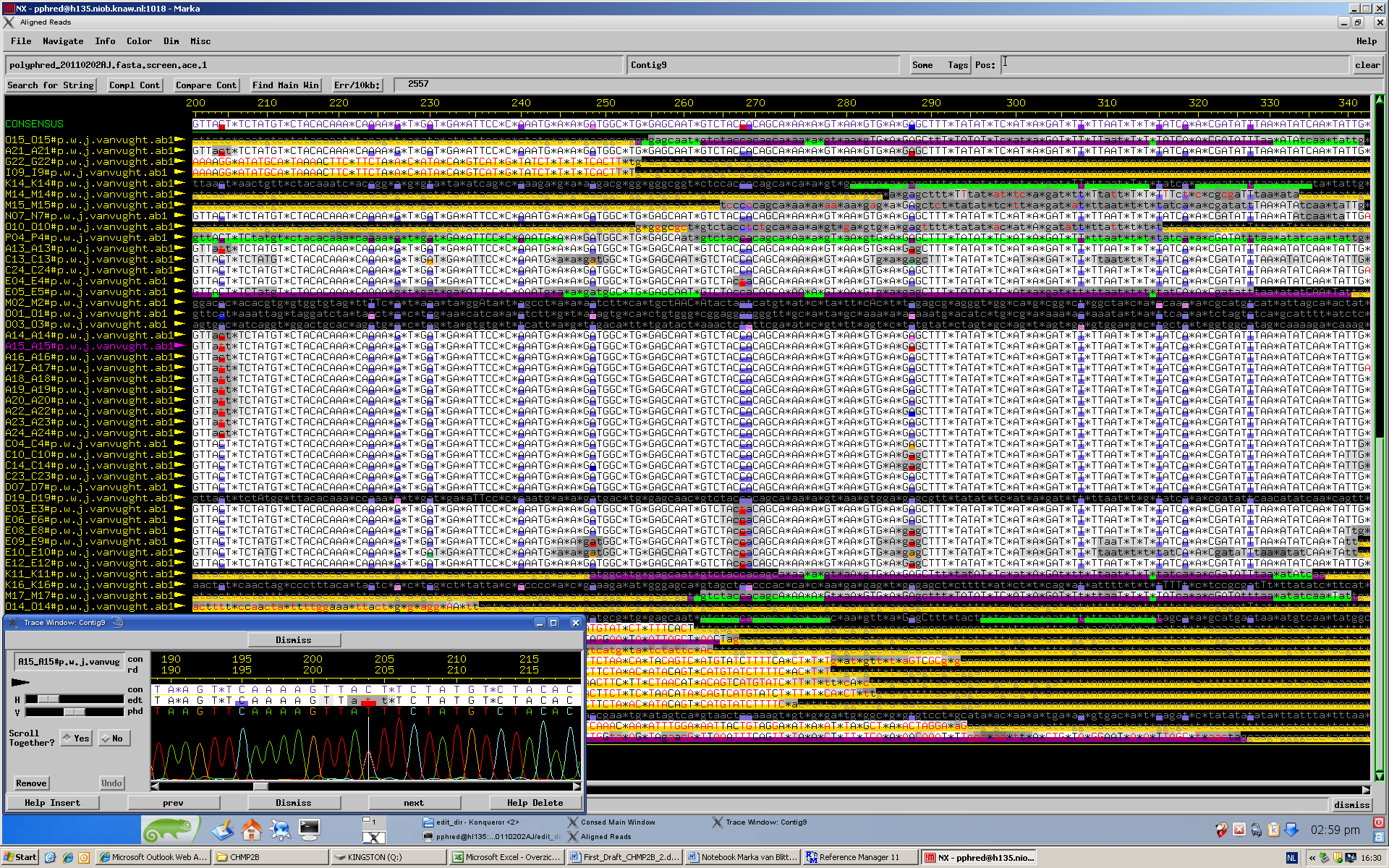


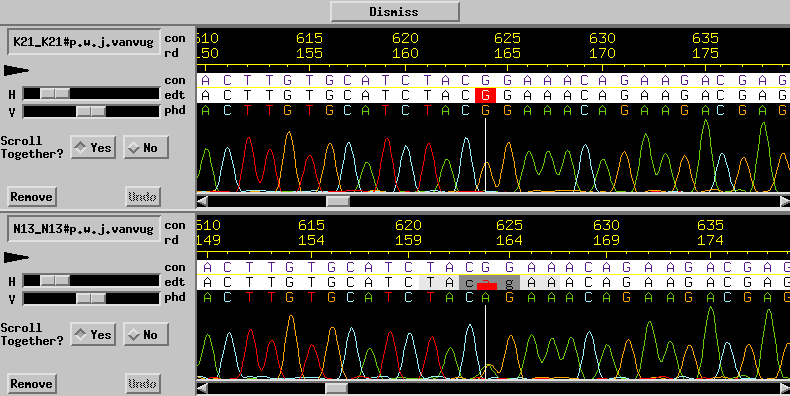


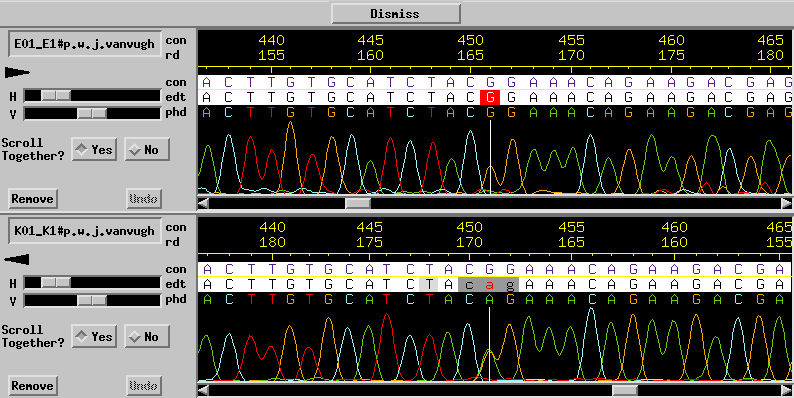


Human V I K E Q N R E L R G T Q K M A K I G N K E A C K V K Q L V H L R K Q K T R T A V S S K V T S M S T Q T

Chimpanzee V I K E Q N R E L R G T Q K M A K I G N K E A C K V K Q L V H L R K Q K T R T A V S S K V T S M S T Q T

Mouse V I K E Q N R E L R G T Q K M A K I G N K E A C R V K Q L V H L R K Q K T R T A V S S K V T S M S T Q T

Cow V I K E Q N R E L R G T Q K M A K I G N K E A C R V K Q L V H L R K Q K T R T A V S S K V T S M S T Q T

Opossum V I K E Q N R E L R G T Q K M A K I G N K E A C R V K Q L V Q L R K Q K T R T A V S S K V T S M S T Q T

Zebrafish I I K E Q S K E L R G T Q K M A K T G N R E A C K I K Q L V Q L R K Q K N R T A V S S K V T S M S T Q T

Conservation of amino-acid residues across species was generated using ClustalW2 online tool, http://www.ebi.ac.uk/Tools/msa/clustalw2/.
