# Supplementary material for: Genetic Overlap between Apparently Sporadic Motor Neuron Diseases
Source: PLoS One. 2012 Nov 14;7(11):e48983. doi: 10.1371/journal.pone.0048983 (PMC3498376; doi:10.1371/journal.pone.0048983)
Supplement: Figure S2 — Pedigree of two PMA patients with p.N352S mutations in TARDBP . (DOC) [file pone.0048983.s002.doc]

**Figure S2.** Pedigree of two PMA patients with p.N352S mutations in *TARDBP*.

3

III

IV

V

VI

I

II

VII

1

2

1

2

2

1

1

2

1

2

3

VIII

4

1

2

1

2

1

1

2

1

2

IX

X

XI

1

2

1

2

1

p.N352S

XII

XIII

3

4

3

4

4

3

3

4

3

4

3

3

4

3

4

3

4

p.N352S

2

4
